# Effect of nursery phase feeding budgets on growth performance and economics in weanling pigs

**DOI:** 10.1093/tas/txag026

**Published:** 2026-02-28

**Authors:** Julian Arroyave, Jordan T Gebhardt, Mike D Tokach, Jason C Woodworth, Joel M DeRouchey, Katelyn N Gaffield, Robert D Goodband, Jorge E Estrada, Eric Parr

**Affiliations:** Department of Animal Sciences and Industry, College of Agriculture, Kansas State University, Manhattan, KS, 66506-0201, United States; Department of Diagnostic Medicine/Pathobiology, College of Veterinary Medicine, Kansas State University, Manhattan, KS, 66506-0201, United States; Department of Animal Sciences and Industry, College of Agriculture, Kansas State University, Manhattan, KS, 66506-0201, United States; Department of Animal Sciences and Industry, College of Agriculture, Kansas State University, Manhattan, KS, 66506-0201, United States; Department of Animal Sciences and Industry, College of Agriculture, Kansas State University, Manhattan, KS, 66506-0201, United States; Department of Animal Sciences and Industry, College of Agriculture, Kansas State University, Manhattan, KS, 66506-0201, United States; Department of Animal Sciences and Industry, College of Agriculture, Kansas State University, Manhattan, KS, 66506-0201, United States; Carthage Veterinary Service Ltd, Carthage, IL, 62321, United States; Carthage Veterinary Service Ltd, Carthage, IL, 62321, United States

**Keywords:** diet complexity, feed budget, nursery pigs, performance

## Abstract

Two experiments were conducted to evaluate the effect of nursery phase feeding budgets on weanling pig growth performance and economics. In Exp. 1, a total of 630 pigs (initially 6.0 ± 0.09 kg) were sorted by body weight (**BW**) and then randomly assigned to treatments arranged in a 3 × 3 factorial under a generalized randomized block design. Main effects included nursery phase feeding budget (high, medium, or low) and BW category (light, medium, or heavy). There were five pigs per pen and 14 pens per interaction mean (42 pens per main effect). The feed budgets for high, medium, and low programs were 1.8, 0.9, and 0 kg/pig for phase 1, and 5.4, 3.6, and 1.8 kg/pig for phase 2, respectively. After the allotted feed budgets were finished, all pigs transitioned to a common diet until day 42. Diets were formulated to provide the same amino acid concentrations but using different ingredients to reduce diet complexity between phases. Phase 1 was formulated with specialty animal protein and lactose products and contained 18.0% lactose with 16.3% soybean meal. In phase 2, specialty protein sources and the lactose level were reduced, with diets containing 7.2% lactose and 22.5% soybean meal. Phase 3 was a common corn-soybean meal-based diet with no specialty protein or lactose sources. In Exp. 1, the response of the budget program was independent of initial BW. Overall, no differences were observed between budget programs for any performance criteria. To validate these results, an experiment was conducted in a commercial research facility. A total of 1170 pigs (initially 5.7 ± 0.32 kg) were used in a 63-d experiment with 26 pigs per pen and 15 pens per treatment. Pigs were randomly assigned to one of three budget programs in a randomized complete block design. Feed budgets were identical to those used in Exp. 1. followed by common diets until d 63. Pigs fed the high-budget program tended (*P* < .10) to have increased daily gain and feed intake during period 2 (d 14 to 33); however, by d 63, no differences between nursery budget programs were observed for any of the response criteria. In both experiments, feed cost and feed cost per kg of gain decreased (*P* < .001) as feed budget was reduced and simpler diets were used. Income over feed cost was maximized (*P* ≤ .004) with the use of the low-budget program in both experiments. In conclusion, reducing phase 1 and 2 budgets improved economic outcomes without affecting overall growth performance.

## Teaser

Reducing phase 1 and 2 nursery feed budgets did not impact overall growth performance and improved economic outcomes.

## Introduction

During the nursery period, three to four dietary phases are typically used (Faccin et al. 2023), with feed budgets allocated according to the average body weight of the group (Mahan et al. 1998). Although dietary strategies may vary depending on ingredient availability, cost, or other criteria of the nutritionist, it is common for early-phase diets to be highly complex (high amounts of lactose and specialty protein sources), with complexity gradually reduced in subsequent phases.

Although chemical composition of diets can be confused with diet complexity, it is important to note that two diets can have similar nutrient profiles and chemical composition, but differ greatly in complexity (number of ingredients in the diets and their physicochemical characteristics; Koo et al. 2017). Complex diets often include specialty ingredients such as dairy products and animal protein sources to enhance palatability and nutrient digestibility, thereby improving feed intake and growth performance immediately after weaning (Mavromichalis et al. 2001; Berrocoso et al. 2012). However, the high ingredient cost associated with these diets has raised questions about their long-term economic benefits, especially considering that improvements in early nursery performance may not persist throughout the grow-to-finish period (Whang et al. 2000; Skinner et al. 2014).

The lack of long-term effects of complex diets could be explained by compensatory growth, a physiological process characterized by an accelerated growth that occurs when animals are provided nutrient-adequate diets following a period of restriction (Hornick et al. 2000; Menegat et al. 2020). Thus, intentional reductions in early dietary complexity or nutrient density may be justified if pigs can achieve compensatory growth in later phases, thereby maintaining overall performance while enhancing economic profitability. Therefore, the objective of this study was to evaluate the effects of reducing the amounts, or budget, of the first two dietary phases on growth performance and economic outcomes in weanling pigs. The hypothesis was that reducing dietary complexity in early phases would not affect overall nursery performance due to the potential for compensatory gain, and that such strategies would improve economic return.

## Materials and methods

### General

The Kansas State University Institutional Animal Care and Use Committee approved the protocols used in these experiments (IACUC # 4485, 4943, and 5117). Experiment 1 consisted of two trials conducted under university conditions. Replicate 1 was conducted between November and December 2023 in two independent rooms within the same barn located at the Kansas State University Swine Teaching Center in Manhattan, KS. Each pen (1.2 × 1.5 m) provided approximately 0.36 m^2^ per pig and was equipped with a 4-hole dry feeder and nipple waterer. Replicate 2 was conducted between December 2024 and January 2025 in two identical barns located at the Kansas State University Segregated Early Weaning facility in Manhattan, KS. Each pen (1.2 × 1.5 m) provided approximately 0.36 m^2^/pig and was equipped with a 4-hole dry feeder and a cup drinker. Facilities were mechanically ventilated with room temperatures set at 32.2°C during the first 10 d and then gradually reduced by 0.28°C daily until the set point reached 25°C.

Experiment 2 was conducted between July and September 2025 in two rooms within the same barn at a commercial weaning-to-finishing facility in Carthage, IL. One room contained 24 pens and the other 21 pens. Each pen (3.0 × 5.8 m) provided approximately 0.67 m^2^/pig and was equipped with a 3-hole dry feeder and two cup drinkers. Room temperature was maintained at a minimum of 28°C during the first week and reduced by 3°C weekly until reaching 22°C. Gas brooders (1 per 2 pens) were used during the first 2 wk after weaning to maintain pig comfort.

### Animals and diets

In Exp. 1, a total of 630 pigs (Line 241 × 600 or 200 × 400; DNA, Columbus, NE) initially weighing 6.0 ± 0.09 kg with an average weaning age of 20 d were used in a 42-d study. Pigs were weaned at approximately 20 d of age and divided into 3 body weight **(BW)** blocks, each containing 33.3% of the pigs. Pigs were then randomly assigned to pens within BW block and pens of pigs were allotted to one of three feed budget programs in a generalized randomized block design arrangement. Each pen had five pigs, and there were 14 replicates per budget program within each weight category for a total of 42 pens per budget program. The two identical rooms within each replicate had an equal representation of dietary treatments and BW blocks. Treatments were arranged in a 3 × 3 factorial with main effects of phase feeding budget (high, medium, or low) and BW category (light, medium, and heavy). Each budget program consisted of different quantities of phases 1 and 2 diets followed by a common phase 3 diet. The feed budgets for high, medium, and low programs were 1.8, 0.9, 0 kg/pig for phase 1 and 5.4, 3.6, and 1.8 kg/pig for phase 2, respectively. All diets were formulated to provide 1.35% standardized ileal digestible **(SID)** Lys with similar amino acid ratios relative to Lys across phases but using different ingredient compositions (Table 1). Phase 1 diets contained specialty animal protein and lactose sources, including spray-dried bovine plasma and spray-dried whey, and were formulated to contain 18.0% lactose and 16.3% soybean meal. Zinc from zinc oxide was added at 3000 mg/kg of Zn to phase 1 diets. In phase 2, spray-dried bovine plasma was removed, lactose was reduced to 7.2%, Zn was reduced to 2000 mg/kg, and soybean meal was increased to 22.5%. Although the chemical composition of phases 1 and 2 were identical between replicates, the specialty soybean ingredient and phytase source differed. In replicate 1, specialty soybean meal (AX3 Digest; Proteka, Newport Beach, CA) and Ronozyme Hiphos 2700 (dsm-firmenich, Parsippany, NJ) were used, whereas enzymatically treated soybean meal (HP 300; Hamlet Protein, Findlay, OH) and HiPhorius 2400 (dsm-firmenich, Parsippany, NJ) were used in replicate 2. The phase 3 diet was identical for both replicates and were conventional corn–soybean meal-based without specialty protein or lactose sources but included 250 mg/kg of Cu from CuSO_4_. All diets were fed in pellet form to avoid any confounding factor in the growth performance responses associated with the feed form (mash vs. pellet). Pigs and feeders were weighed on d 0, 10, 24, and 42 to determine average daily gain (**ADG**), average daily feed intake (**ADFI**), and gain: feed ratio (**G: F**). Fecal samples were collected via rectal palpation from the same three pigs per pen on d 10 and 24 in replicate 1, but not in replicate 2, to determine fecal dry matter (**DM**).

**Table 1.** Diet composition Exp. 1 (as-fed basis)^a^

Item	Phase 1		Phase 2		Phase 3
Ingredient, %	Rep. 1	Rep. 2	Rep. 1	Rep. 2	–
** Corn**	44.99	44.12	57.58	56.42	62.27
** Soybean meal, 47.7 % CP**	16.34	16.29	22.43	22.49	32.66
** Spray-dried whey**	25.00	25.00	10.00	10.00	–
** Spray-dried bovine plasma**	2.50	2.50	–	–	–
** Specialty soybean meal^b^**	5.00	–	5.00		–
** Enzymatically treated soybean meal^c^**	–	6.00	–	6.00	–
** Soybean oil**	3.00	3.00	1.00	1.00	1.00
** Limestone**	0.65	0.65	0.74	0.74	0.82
** Monocalcium phosphate**	0.50	0.48	1.00	0.98	1.10
** Salt**	0.30	0.23	0.50	0.56	0.60
** L-Lys-HCl**	0.37	0.38	0.49	0.49	0.48
** DL-Met**	0.22	0.22	0.21	0.21	0.20
** L-Thr**	0.16	0.17	0.23	0.23	0.23
** L-Trp**	0.03	0.03	0.04	0.04	0.04
** L-Val**	0.07	0.08	0.11	0.12	0.11
** Trace mineral premix^d^**	0.15	0.15	0.15	0.15	0.15
** Vitamin premix^e^**	0.25	0.25	0.25	0.25	0.25
** Phytase^f^**	0.03	0.03	0.03	0.03	0.03
** Choline chloride 60%**	0.05	0.05	–	–	–
** Zinc oxide**	0.39	0.39	0.25	0.25	–
** Copper sulfate**	–	–	–	–	0.07
**Total**	100.00	100.00	100.00	100.00	100.00
**Calculated analysis**					
**Standardized ileal digestible amino acids, %**					
** Lys**	1.35	1.35	1.35	1.35	1.35
** Ile: Lys**	57	57	57	57	57
** Leu: Lys**	117	117	115	115	116
** Met: Lys**	36	36	36	36	36
** Met & Cys: Lys**	59	59	57	57	57
** Thr: Lys**	65	65	65	65	65
** Trp: Lys**	20	20	20	20	20
** Val: Lys**	69	69	69	69	69
** His: Lys**	34	34	35	35	37
**NE, kcal/kg**	2,643	2,643	2,502	2,502	2,454
**CP, %**	20.4	20.3	20.9	20.8	21.6
**Lactose, %**	18.0	18.0	7.2	7.2	–
**Ca, %**	0.63	0.67	0.68	0.72	0.69
**P, %**	0.57	0.58	0.62	0.63	0.63
**STTD P, %^g^**	0.51	0.51	0.51	0.51	0.48

a Two replicate experiments were conducted; specialty soybean ingredient and phytase source differed between replicates, but the chemical composition of each dietary phase was the same across replicates. Phases 1, 2, and 3 were fed according to the treatment structure in pellet form.

b AX3 Digest (Proteka, Newport Beach, CA).

c HP 300; Hamlet Protein; Findlay, OH.

d Provided per kilogram of feed: 110 mg of Zn as zinc sulfate; 17 mg of Cu as copper sulfate; 33 mg of Mn as manganese oxide; 110 mg of Fe as ferrous sulfate; 0.30 mg of I as ethylenediamine dihydriodide; 0.30 mg of Se as sodium selenite.

e Provided per kilogram of feed: 4134 IU of vitamin A; 1653 IU of vitamin D; 44 IU of vitamin E; 3.31 mg of vitamin K3; 33 µg of vitamin B_12_; 8 mg of riboflavin; 28 mg of pantothenic acid; 50 mg of niacin.

f Repetition 1 included Ronozyme Hiphos 2700 (dsm-firmenich, Parsippany, NJ) and repetition 2 included HiPhorius 2400 (dsm-firmenich, Parsippany, NJ). Both phytase sources were included at 811 FYT/kg with an assumed release of 0.11% STTD P across all dietary phases.

g Standardized total tract digestible P.

For Exp. 2, a total of 1170 pigs (PIC 337 × 1050, Hendersonville, TN) initially weighing 5.7 ± 0.32 kg with an average weaning age of 22.6 d were used in a 63-d experiment with 26 pigs per pen (13 gilts and 13 barrows) and 15 pens per treatment. Pigs were assigned to pens in groups of six pens. Briefly, 39 pigs of a single sex were weighed together to estimate the average BW of the group. The 3 heaviest and 3 lightest pigs were visually identified and marked using different paint spray colors. The 39 pigs were then divided into six subgroups, with each subgroup containing 1 heavy and 1 light pig. The remaining pigs were subsequently distributed among the subgroups to ensure that the average BW of each subgroup matched the group mean. This procedure was repeated for pigs of both sexes, after which one subgroup of barrows and one subgroup of gilts were combined to form a pen. Feed budget treatments were the same as described for Exp. 1. Diets in Exp. 2 closely matched those in replicate 2 of Exp. 1, except for different vitamin and mineral premixes, phytase, and fat sources. Because pigs in Exp. 2 were housed in a wean-to-finish facility, performance was monitored until d 63 post-placement. At d 42, corresponding to the end of the nursery phase, all pigs were transitioned to a common grower diet (phase 4) provided in mash form (Table 2). Similar to Exp. 1, phases 1, 2, and 3 diets were provided in pellet form.

**Table 2.** Diet composition Exp. 2 (as-fed basis)^a^

Item	Phase 1	Phase 2	Phase 3	Phase 4
**Ingredient, %**				
** Corn**	44.19	56.48	62.35	61.72
** Soybean Meal, 47.0 % CP**	16.28	22.54	32.66	21.10
** Corn DDGS, 7% oil**	–	–	–	14.25
** Spray-dried bovine plasma**	2.50	–	–	–
** Spray-dried whey**	25.00	10.00	–	–
** Enzymatically treated soybean meal^b^**	6.00	6.00	–	–
** Corn oil**	3.00	1.00	1.00	–
** Calcium carbonate**	0.76	0.86	0.93	1.03
** Monocalcium phosphate**	0.48	0.98	1.10	0.26
** Salt**	0.23	0.56	0.60	0.54
** L-Lys-HCl**	0.38	0.49	0.48	0.52
** DL-Met**	0.22	0.21	0.20	0.14
** L-Thr**	0.17	0.23	0.23	0.19
** L-Trp**	0.03	0.04	0.04	0.05
** L-Val**	0.08	0.12	0.11	0.07
** Choline chloride, 60%**	0.05	–	–	–
** Zinc oxide**	0.39	0.25	–	–
** Copper sulfate**	–	–	0.07	–
** VTM with phytase^c^**	0.25	0.25	0.25	0.15
**Total**	100.00	100.00	100.00	100.00
**Calculated analysis**				
**Standardized ileal digestible amino acids, %**				
** Lys**	1.35	1.35	1.35	1.15
** Ile: Lys**	57	57	57	57
** Leu: Lys**	115	113	116	137
** Met: Lys**	36	36	36	37
** Met & Cys: Lys**	59	57	57	59
** Thr: Lys**	65	65	65	65
** Trp: Lys**	19.7	19.7	19.6	19.2
** Val: Lys**	69	69	69	71
** His: Lys**	34	35	37	39
**NE, kcal/kg**	2,654	2,500	2,460	2,457
**CP, %**	20.3	20.8	21.6	19.8
**Lactose, %**	18.0	7.2	–	–
**Ca, %**	0.66	0.72	0.72	0.54
**P, %**	0.58	0.63	0.63	0.45
**STTD P, %^d^**	0.51	0.51	0.48	0.34

1 Phases 1 and 2 were fed according to the treatment structure in pellet form, followed by a common phase 3 diet in pellet form until d 42, and a common phase 4 diet in mash form between d 42 and 63.

2 HP 300; Hamlet Protein; Findlay, OH.

3 Vitamin and trace mineral premix, including 750 FTU/kg of phytase (Quantum Blue, ABVista, Plantation, FL) with an assumed release of 0.11% STTD P. Provided per kilogram of feed: Phases 1 to 3: 120 mg of Zn as zinc sulfate; 16 mg of Cu as copper sulfate; 45 mg of Mn as manganese oxide; 100 mg of Fe as ferrous sulfate; 0.70 mg of I as ethylenediamine dihydriodide; 0.30 mg of Se as sodium selenite; 9000 IU of vitamin A; 2000 IU of vitamin D; 72 IU of vitamin E; 4 mg of vitamin K3; 40 µg of vitamin B_12_; 9 mg of riboflavin; 30 mg of pantothenic acid; 44 mg of niacin. Phase 4: 120 mg of Zn as zinc sulfate; 150 mg of Cu as tribasic copper chloride; 35 mg of Mn as manganese oxide; 100 mg of Fe as ferrous sulfate; 0.4 mg of I as ethylenediamine dihydriodide; 0.30 mg of Se as sodium selenite; 2866 IU of vitamin A; 838 IU of vitamin D; 31 IU of vitamin E; 3 mg of vitamin K3; 26 µg of vitamin B_12_; 6 mg of riboflavin; 22 mg of pantothenic acid; 22 mg of niacin.

4 Standardized total tract digestible P.

In Exp. 2, daily feed additions were recorded using a computerized feeding system (DryExtra Pro, Big Dutchman, Holland, MI). Pigs were weighed, and the amount of feed remaining in the feeders was estimated based on feeder volume using a regression equation on d 0, 7, 14, 21, 33, 42, and 63 to calculate ADG, ADFI, and G: F. On d 42, after pen weights were collected, individual pigs were weighed to determine the coefficient of variation of pig weights within each pen. Pigs removed from experimental pens due to health problems or slow growth were ear-tagged with a consecutive number and placed in independent pens within each room, where they received special care. At the end of the experiment, the remaining pigs in these pens were counted to determine survivability. Fecal samples were collected via rectal palpation from five pigs per pen on d 14 of the study. These samples were stored at −4°C until fecal dry DM analysis was conducted. The samples were dried in a forced-air oven for 48 h at 55°C for the determination of fecal DM.

No feed-grade medications were added for any of the dietary phases; however, because pigs came from a porcine reproductive and respiratory syndrome (PRRS) virus positive flow in Exp. 2, water medications and acidifiers were used in both barns according to the following program and administered via drinking water. Gentamicin sulfate (Gen-Gard, Huvepharma, Peachtree City, GA) was used from d 1 to 5, followed by trimethoprim/sulfadiazine (Equisul, Aurora Pharmaceutical, Northfield, MN) from d 6 to 10, Tyvalosin (Aivlosin, Pharmgate Animal Health, Wilmington, NC) from d 11 to 15, citric acid (citric acid, Zinpro, Rainsville, AL) from d 12 to 19, amoxicillin (Ultramox, Veterinary Solutions, St. Peters, MN) from d 20 to 25, and trimethoprim/sulfadiazine (Equisul, Aurora Pharmaceutical, Northfield, MN) from d 31 to 35. Antibiotics doses were based on veterinary recommendations and citric acid dose was set based on manufacturer recommendations.

### Economic analysis

Total feed cost per pig, feed cost/kg of gain, revenue, and income over feed cost (**IOFC**) were calculated. Feed cost per pig placed was determined by multiplying total feed intake by diet cost. Feed cost/kg gain was calculated by dividing the total feed cost per pig by the total weight gained (including mortality and removals). The total cost for each phase, including feedmill and delivery fees, was $0.63, 0.44, and 0.34 per kg for phases 1, 2, and 3, respectively, in Exp. 1, and $0.84, 0.51, 0.30, and 0.25 per kg for phases 1, 2, 3, and 4, respectively, in Exp. 2. Revenue was calculated based on a carcass price of $1.81/kg, and an assumed carcass yield of 75%.

### Statistical analysis

Data were analyzed using RStudio [Version 4.0.2 (2020-06-22), R Core Team, R Foundation for Statistical Computing, Vienna, Austria] with pen serving as the experimental unit. For performance and economic variables, in Exp. 1, the model included phase-feeding budget, BW category, and their interaction as fixed effects, with barn (1 to 4) as a random effect. For Exp. 2, the model included phase-feeding budget as a fixed effect, with room and BW block nested within room as random effects.

For fecal DM, in addition to the performance criteria included in each model within each experiment, pen was included as a random effect to account for subsampling associated with multiple individual pigs analyzed from each pen. Mortality, removals, and total mortality and removals data were analyzed using a binomial distribution. The proportion of dead or removed pigs over the initial pigs placed per pen was used as the response variable for mortality and removals. Results were considered significant with *P* ≤ 0.05 and marginally significant with *P* ≤ 0.10.

## Results

### Experiment 1

Overall, pigs were healthy with no mortality or removals. No interactions were observed between the budget program and BW category on growth performance and economics; therefore, analyses focused on the main effect of budget program and BW category.

For the main effect of the budget program, during period 1 (d 0 to 10), no differences were observed between feed budget programs for d 10 BW and ADG (Table 3). There was a tendency (*P* = 0.075) for increased ADFI with increasing phase 1 and 2 feed budgets; however, no mean separation was observed. Consequently, G:F increased (*P* < 0.05) in the low-budget program compared with the medium program, with the high program intermediate. For period 2 (d 10 to 24), no differences were observed between pigs provided the different feed budgets for any of the performance criteria. In period 3 (d 24 to 42), pigs previously fed the high-budget program had decreased (*P* = 0.05) ADG than those fed the medium program, whereas no differences were observed for ADFI. As a result, G:F tended (*P* = 0.078) to increase as phase 1 and 2 budgets were reduced, although no mean separation was observed. At the end of the experiment on d 42, BW tended to decrease with the use of high-budget program, although no mean separation was observed between treatments. No differences were observed for overall ADG, ADFI, or G:F.

**Table 3.** Effect of phase feeding budget on nursery performance, fecal dry matter, and economics, Exp. 1^1^

	Feed budget, kg/pig^2^				
	High	Medium	Low		
			
Phase 1:	1.8	0.9	0		
Phase 2:	5.4	3.6	1.8	SEM	*P* =
**BW, kg**					
** d 0**	5.9	6.0	6.0	0.09	0.407
** d 10**	7.3	7.3	7.4	0.26	0.755
** d 24**	13.5	13.6	13.7	0.67	0.468
** d 42**	25.5	26.0	26.0	1.20	0.060
**Period 1 (d 0 to 10)**					
** ADG, g**	142	139	143	20.1	0.709
** ADFI, g**	178	171	166	5.3	0.075
** G:F, g/kg**	799^ab^	784^b^	852^a^	105.0	0.011
**Period 2 (d 10 to 24)**					
** ADG, g**	443	445	452	30.0	0.617
** ADFI, g**	541	545	551	39.7	0.624
** G:F, g/kg**	822	816	822	11.9	0.766
**Period 3 (d 24 to 42)**					
** ADG, g**	685^a^	712^b^	703^ab^	19.8	0.013
** ADFI, g**	923	947	934	38.0	0.129
** G:F, g/kg**	744	754	755	11.7	0.078
**Overall (d 0 to 42)**					
** ADG, g**	473	483	483	24.2	0.137
** ADFI, g**	615	624	619	32.3	0.533
** G:F, g/kg**	770	775	780	4.8	0.103
**Fecal DM, %^3^**					
** d 10**	20.4^ab^	20.1^a^	23.1^b^	0.905	0.018
** d 24**	18.8	16.6	18.7	0.905	0.111
**Economics, $/pig placed^4^**					
** Feed cost**	9.45^a^	9.01^b^	8.48^c^	0.756	< 0.001
** Feed cost/kg of gain^5^**	0.48^a^	0.45^b^	0.42^c^	0.013	< 0.001
** Revenue^6^**	26.92	27.30	27.43	1.375	0.361
** IOFC^7^**	17.47^a^	18.29^b^	18.95^c^	0.636	< 0.001

1 Two replicate experiments were conducted using a total of 630 pigs (Line 241 × 600 or 200 × 400; DNA, Columbus, NE; initial BW = 6.0 ± 0.09 kg) in a 42-d growth study with 5 pigs per pen and 42 pens per treatment. There were no removals or mortalities in the experiment.

2 After receiving their assigned phase 1 and 2 diets according to experimental treatments, pigs were fed a common phase 3 diet until d 42 of the experiment.

3 Fecal dry matter was only estimated in one of the experiments. Fecal samples were collected via rectal palpation from the same 3 pigs per pen on d 10 and 24.

4 Feed cost per kg feed was: Phase 1 = $0.63; Phase 2 = $0.44; Phase 3 = $0.34.

5 Feed cost/kg of gain = total feed cost ÷ total gain per pen.

6 Revenue = (total gain/pig placed × 0.75) × $1.81.

7 Income over feed cost = revenue—feed cost.

On d 10, pigs fed with the low program had increased fecal DM (*P* < 0.05) compared to those fed the medium-budget program, with pigs fed the high-budget program intermediate. However, no differences between budget programs were observed for d 24 fecal DM.

Feed cost per pig and feed cost/kg of gain decreased (*P* < 0.001) as the phase feeding budgets were decreased. No differences were observed for revenue, resulting in an improvement (*P* < 0.001) in IOFC as the phase feeding budgets decreased.

For the main effect of weaning BW category, as expected, BW was different (*P* < 0.001) between the three BW categories on d 0, 10, 24, and 42 (Table 4). For period 1 (d 0 to 10), light pigs had decreased (*P* < 0.05) ADG and ADFI than the other two BW categories. However, no differences were observed for G:F. In period 2 (d 10 to 24), period 3 (d 24 to 42), and overall (d 0 to 42), ADG and ADFI increased (*P* < 0.001) as initial BW increased. In period 2 (d 10 to 24), medium pigs tended (*P* = .091) to have decreased G:F compared with the other two BW categories. However, no differences in G:F were observed between any BW category in period 3 (d 24 to 42) and overall (d 0 to 42). On d 10, light pigs had increased fecal DM (*P* < 0.05) compared to medium pigs. However, no differences between BW categories were observed for d 24 fecal DM. Feed cost, revenue, and IOFC increased (*P* < 0.001) as initial BW increased. However, no differences between BW categories were observed for feed cost/kg of gain.

**Table 4.** Effect of body weight on nursery performance and economics, Exp. 1^1^

	BW category^2^				
Item	Light	Medium	Heavy	SEM	*P* =
**BW, kg**					
** d 0**	4.9^a^	5.9^b^	6.9^c^	0.09	< 0.001
** d 10**	6.2^a^	7.4^b^	8.9^c^	0.26	< 0.001
** d 24**	12.0^a^	13.6^b^	15.2^c^	0.67	< 0.001
** d 42**	25.3^a^	26.0^b^	28.2^c^	1.20	< 0.001
**Period 1 (d 0 to 10)**					
** ADG, g**	129^a^	149^b^	145^b^	20.2	0.001
** ADFI, g**	158^a^	178^b^	180^b^	5.3	< 0.001
** G:F, g/kg**	798	834	804	105.1	0.242
**Period 2 (d 10 to 24)**					
** ADG, g**	414^a^	440^b^	486^c^	30.0	< 0.001
** ADFI, g**	502^a^	547^b^	587^c^	39.7	< 0.001
** G:F, g/kg**	826	807	826	12.0	0.091
**Period 3 (d 24 to 42)**					
** ADG, g**	652^a^	710^b^	740^c^	19.8	< 0.001
** ADFI, g**	868^a^	945^b^	991^c^	38.1	< 0.001
** G:F, g/kg**	753	752	747	11.7	0.450
**Overall (d 0 to 42)**					
** ADG, g**	445^a^	483^b^	510^c^	24.2	< 0.001
** ADFI, g**	573^a^	625^b^	659^c^	32.3	< 0.001
** G:F, g/kg**	778	773	774	4.8	0.490
**Fecal DM, %^3^**					
** d 10**	22.6^b^	19.4^a^	21.7^ab^	0.905	0.018
** d 24**	18.1	17.7	18.3	0.905	0.111
**Economics, $/pig placed^4^**					
** Feed cost**	8.36^a^	9.07^b^	9.52^c^	0.756	< 0.001
** Feed cost/kg of gain^5^**	0.45	0.45	0.45	0.013	0.340
** Revenue^6^**	25.24^a^	27.42^b^	28.98^c^	1.375	< 0.001
** IOFC^7^**	16.88^a^	18.36^b^	19.47^c^	0.636	< 0.001

1 A total of 630 pigs (Line 241 × 600 or 200 × 400; DNA, Columbus, NE; initial BW = 6.0 ± 0.09 kg with an average weaning age of 21 d) were used in a 42-d growth study with 5 pigs per pen and 42 pens per treatment.

2 At placement pigs were divided into three BW categories each containing 33.33% of the pigs.

3 Fecal dry matter was only estimated in one of the experiments. Fecal samples were collected via rectal palpation from the same 3 pigs per pen on d 10 and 24.

4 Feed cost per kg feed was: Phase 1 = $0.63; Phase 2 = $0.44; Phase 3 = $0.34. Calculations were based on the total number of pigs placed (5 pigs/pen).

5 Feed cost/kg of gain = total feed cost ÷ total gain per pen.

6 Revenue = (total gain/pig placed × 0.75) × $1.81.

7 Income over feed cost = revenue—feed cost.

### Experiment 2

For period 1 (d 0 to 14), no differences were observed for d 14 BW, ADG, and ADFI (Table 5). However, pigs fed the low-budget program tended (*P* = 0.059) to have increased G:F compared with those fed the high-budget program, with pigs fed the medium-budget program intermediate. For period 2 (d 14 to 33), pigs fed the high-budget program tended (*P* = 0.074) to have increased ADG; however, no mean separation between treatments was observed. No differences were observed for d 33 BW, ADFI, or G:F. For period 3 (d 33 to 42), period 4 (d 42 to 63), and the overall study (d 0 to 63), no differences were observed for any of the performance criteria. For body weight coefficient of variation on d 42, no differences were observed among treatments. No differences in mortality, removals, or total removals were observed among feed budget programs; however, pigs fed the high budget program exhibited numerically lower mortality and removals compared with those fed the medium or low budget programs.

**Table 5.** Effect of phase feeding budget on nursery performance and economics, Exp. 2^1^

	Feed budget, kg/pig^2^				
	High	Medium	Low		
			
Phase 1:	1.8	0.9	0		
Phase 2:	5.4	3.6	1.8	SEM	*P* =
**BW, kg**					
** d 0**	5.7	5.7	5.7	0.32	0.488
** d 14**	9.0	9.0	9.1	0.79	0.710
** d 33**	19.0	18.6	18.8	0.98	0.396
** d 42**	24.7	24.3	24.5	1.12	0.326
** d 63**	40.1	39.6	40.1	1.76	0.346
**Period 1 (d 0 to 14)**					
** ADG, g**	237	235	243	34.5	0.755
** ADFI, g**	286	275	276	34.4	0.449
** G:F, g/kg**	827^a^	851^ab^	879^b^	21.9	0.059
**Period 2 (d 14 to 33)**					
** ADG, g**	522	501	505	11.6	0.074
** ADFI, g**	663	648	647	20.7	0.242
** G:F, g/kg**	703	696	701	6.3	0.532
**Period 3 (d 33 to 42)**					
** ADG, g**	634	619	627	15.6	0.444
** ADFI, g**	997	973	980	28.4	0.243
** G:F, g/kg**	636	636	639	5.4	0.903
**Period 4 (d 42 to 63)**					
** ADG, g**	735	730	744	1.1	0.435
** ADFI, g**	1336	1316	1332	48.2	0.579
** G:F, g/kg**	550	555	559	5.2	0.329
**Overall (d 0 to 63)**					
** ADG, g**	544	531	541	23.6	0.126
** ADFI, g**	848	827	834	34.1	0.167
** G:F, g/kg**	642	643	649	3.7	0.350
** Mortality, %**	1.02	2.55	2.04	0.876	0.293
** Removals, %**	1.00	1.82	1.41	0.758	0.562
** Total removals and mortality, %**	2.18	4.61	3.63	1.158	0.170
**Coefficient of variation, %^3^**					
** d 42**	17.41	17.91	17.61	1.168	0.904
**Fecal dry matter, %^4^**					
** d 14**	22.62	23.09	20.49	0.756	0.046
**Economics, $/pig placed^5^**					
** Feed cost**	17.19^a^	15.86^b^	15.16^c^	1.030	<0.001
** Feed cost/kg of gain^6^**	0.51^a^	0.49^b^	0.46^c^		<0.001
** Revenue^7^**	45.87^a^	44.15^b^	45.23^ab^	2.159	0.049
** IOFC^8^**	28.68^a^	28.29^a^	30.07^b^	1.141	0.004

1 A total of 1170 pigs (PIC 337 × 1050, Hendersonville, TN; initial BW = 5.7 ± 0.32 kg with an average weaning age of 22.6 d) were used in a 63-d study with 26 pigs per pen and 15 pens per treatment. Pigs were placed in two independent rooms within the same barn, in a wean-to-finish commercial facility located in Carthage, IL.

2 The phase-feeding budget consisted of varying quantities of phase 1 and 2, followed by a common phase 3 diet until d 42 and a common phase 4 diet until d 63 of the experiment.

3 At d 42, all pigs were individually weighed to estimate the coefficient of variation for pig weight within each pen.

4 Fecal samples were collected from 5 pigs per pen.

5 Feed cost per kg feed was: Phase 1 = $0.84; Phase 2 = $0.51; Phase 3 = $0.30; and Phase 4 = $0.25. Calculations were based on the total number of pigs placed (26 pigs/pen).

6 Feed cost/kg of gain = total feed cost ÷ total gain per pen.

7 Revenue = (total gain/pig placed × 0.75) × $1.81.

8 Income over feed cost = revenue—feed cost.

A treatment effect was detected (*P* = 0.046) for fecal DM at d 14, where pigs fed the low-budget program had numerically lower fecal DM compared with pigs fed the medium or high budget programs; however, no pairwise differences were observed among treatments when using a Tukey multiple comparison adjustment.

Feed cost and feed cost per kg of gain decreased (*P* < 0.001) as phases 1 and 2 feed budgets were reduced. Revenue from pigs fed the high-budget program was greater (*P* < 0.05) than those fed the medium-budget program; however, no differences were observed between the low-budget and the other programs. As a result, the use of the low-budget program resulted in greater (*P* < 0.05) IOFC than high- or medium-budget programs.

## Discussion

Feed cost accounts for the largest proportion of total production cost in the swine industry (Alves et al. 2022); therefore, developing nutritional strategies that optimize profitability has been a long-standing area of research. The initial dietary phases are typically the most expensive because they contain highly digestible ingredients and feed additives that facilitate the transition from a liquid- to solid feed (Carnino et al. 2025). Although complex diets are widely used in the swine industry, several reports have indicated that the benefits observed during the early nursery phase may not carry-over during the late nursery or grower periods due to compensatory growth (Skinner et al. 2014; Koo et al. 2017). As a result, reducing the allocation of the most expensive diets may lower feed cost without negatively affecting overall pig growth performance. Therefore, the present study was designed to evaluate, across two experiments, the effects of reducing feed budgets in phases 1 and 2 on growth performance and economics.

Although the experiment conducted under university conditions observed no differences in growth performance and improvement in economics as nursery feed budgets were reduced, it remained uncertain whether similar responses would be achieved under commercial production systems. Well-controlled university research facilities generally minimize sources of variation (Magowan et al. 2007). Therefore, Exp. 2 was designed to confirm whether the responses observed under university conditions could be validated in commercial facilities.

Decreasing the time between weaning and initial feed consumption is critical for supporting gut development and reducing post-weaning stress (Carnino et al. 2025). Newly weaned pig often consume little feed due to social stress, changes in feed form (from liquid to solid-based feed), and immature digestive capacity, which slows adaptation to solid feed (Pluske et al. 1997; Xiong et al. 2019). Therefore, strategies to increase palatability and digestibility of nursery diets are essential to shorten the lag in feed intake and minimize the negative impacts of weaning stress on piglet health and performance.

One nutritional approach to facilitate the transition from a liquid to a solid-based diet is the use of highly digestible ingredients in nursery diets (complex diets; Carnino et al. 2025). Readily digestible carbohydrates and protein sources reduce the flow of undigested substrates into the hindgut, thereby lowering microbial fermentation and the associated release of endotoxins that can trigger immune activation (Heo et al. 2009).

Despite there being physiological support behind the use of complex diets in weanling pigs, their effects on growth performance remain inconsistent (Carnino et al. 2025), with much of the observed benefit associated with improvements in ADFI rather than sustained effects on ADG. Several studies have demonstrated that diets containing highly digestible protein and carbohydrate sources stimulate greater feed intake in the immediate post-weaning period (Sulabo et al. 2010; Wang et al. 2018; Christensen et al. 2023). However, improvements in feed intake do not always translate into better growth performance across nursery phases (Koo et al. 2017; Guo et al. 2022; Christensen et al. 2023). Additionally, some reports have indicated that the use of complex diets may contribute to reductions in removals and mortality (Collins et al. 2017) similar to the numerical differences in favor of the high-budget program observed in Exp. 2. Conversely, other studies have observed that feeding complex diets can improve performance not only during the nursery period but also into subsequent production phases (Christensen and Huber 2021).

Similar to the results herein, decreased G: F in the early nursery period associated with the use of complex diets has been previously reported (Bikker et al. 2004; Sulabo et al. 2010; Koo et al. 2017). In all cases, the reduction in G: F was driven by greater feed intake of pigs fed complex diets, while ADG remained unchanged. Also, the reduction of ADG observed in pigs fed the high budget program (longer time fed the complex diet) in Exp. 1 may be explained by alterations in the intestinal microbiome, as the transition from phase 2 to phase 3 occurred for most pigs during period 3 (d 24 to 42). It is well established that complex diets enhance microbial diversity and promote the growth of beneficial bacteria (Frese et al. 2015; Gresse et al. 2017). These microbial communities contribute to intestinal health by producing short-chain fatty acids, lowering gut pH, and preventing the proliferation of opportunistic pathogens (Peng and Biswas 2017). However, when pigs are abruptly transitioned to simple diets based primarily on corn and soybean meal, the reduced availability of easily fermentable substrates may lead to a decline in these beneficial bacterial populations and a transient dysbiosis (Guevarra et al. 2019; Meng et al. 2020). Such disturbances in microbial equilibrium can decrease nutrient digestion and absorption, increase intestinal inflammation, and ultimately contribute to reduced growth performance despite adequate feed intake. Regardless, these mechanisms possibly explain why pigs on the high-budget program exhibited decreased ADG during the later nursery phase in Exp. 1. The lack of a performance response observed in Exp. 2 during the same period may suggest that, under commercial conditions where pigs are exposed to additional uncontrolled factors, the impact of intestinal microbiome alterations may be less evident or masked by other factors.

In Exp. 2, the use of complex diets tended to improve ADG and ADFI during the early nursery period, but this difference was not sustained by d 63. These results align with reports by Whang et al. (2000) and Skinner et al. (2014) where the use of complex diets improved growth performance after weaning, but the differences were lost at the end of the nursery or grower period. Skinner et al. (2014) attributed this response to compensatory gain observed in pigs fed simple diets later in the nursery period.

In most studies, the effect of diet complexity has typically been evaluated using two or more diets that differ in complexity, changing dietary chemical composition between phases, and fed on a fixed time basis. In contrast, the present study employed a budget-based approach, where diet complexity varied across phases, but the SID Lys, amino acid ratios, and macro-minerals were maintained across phases to avoid confounding factors in the performance responses associated with changes in dietary Lys concentration. Under this framework, pigs in the high-budget program received complex diets for a longer duration, whereas the feeding period of complex diets was shortened in the medium- and low-budget program. However, because concentrations of Zn, Cu, lactose, net energy, and crude protein also differed between phases, the responses observed in this study cannot be attributed solely to dietary complexity but rather reflect the combined influence of both complexity and the chemical composition of diets across phases. This approach has also been adopted in other studies, which reported similar responses to those observed in the current study. Lee et al. (2014) evaluated the effect of varying feeding durations of phases 1, 2, and 3 diets in pigs raised under commercial conditions. Their results indicated that, by the end of the nursery period, simplifying the feeding program did not affect final BW or ADG, although extended feeding of complex diets was associated with increased ADFI. Similarly, González-Solé et al. (2024) compared feeding strategies in which pre-starter diets were provided for a fixed duration or until pigs reached a target BW. Their results indicated that extending the feeding of complex pre-starter diets did not improve final growth performance or within-pen uniformity but did reduce mortality among the smallest pigs. Together, these results indicate that reducing the budget for the first dietary phases has minimal impact on overall growth performance; however, they also suggest that factors such as initial BW may influence the response to the feed budget program.

Body weight at birth or weaning is one of the major factors influencing growth performance in pigs. At a given age, heavier pigs at weaning tend to maintain this weight advantage through to marketing (Douglas et al. 2013). Similar to results in Exp. 1, Main et al. (2004), and Collins et al. (2017) did not find a significant interaction between BW at weaning and diet complexity when growth performance of nursery pigs was evaluated. When considering the main effect of initial BW, the greater ADG and ADFI observed as the initial BW increased in Exp. 1 is consistent with studies by Collins et al. (2013), Douglas et al. (2013), and Collins et al. (2017). The better performance of the heavy pigs could be explained by greater maturation of the gastrointestinal tract (Michiels et al. 2013), which allows for improvements in digestive capacity and nutrient absorption. Additionally, differences in muscle fiber composition likely contribute to these growth advantages (Choi et al. 2013).

Multiple reports have indicated that the use of complex diets improved diarrhea score (Berrocoso et al. 2012; Koo et al. 2017), which aligns with the greater fecal DM observed when pigs were fed with the high budget program in Exp. 2. As previously indicated, the use of highly digestible ingredients may promote the growth of beneficial microbiome in the gastrointestinal tract, and reduces the amount of nutrients that escape into the large intestine, which together may contribute to improving the consistency of fecal DM. In contrast, during Exp. 1, the highest d-10 fecal DM was observed with the low-budget program. This discrepancy can be explained by differences in the timing of the phase change. Due to lower ADFI in Exp. 1, the phase change between phases 1 and 2 for the high- and medium-budget programs occurred around d 10, meaning that restructuring of the intestinal microbiome associated with diet changes could have contributed to the lower fecal DM observed.

Highly digestible ingredients, such as dairy products or animal protein sources, are generally expensive, which explains why the initial dietary phases are the most expensive diets pigs are fed. The economic outcomes from both experiments agree with Collins et al. (2017), who observed decreases in feed cost and feed cost per kg gain when simple diets were used. However, the differences observed across experiments for revenue and IOFC responses to budget programs were unexpected. Because different feed budgets did not affect overall performance and feed cost decreased with the use of simpler programs, it was anticipated that revenue would remain unchanged and IOFC would improve as the amount of phase 1 and 2 diets was reduced, as observed in Exp. 1. However, in Exp. 2, numerical differences in final BW among treatments influenced revenue calculations. Specifically, pigs fed the medium-budget program had lower numerical BW gain, likely because this group was more affected by health challenges, as reflected by greater mortality. It is important to recognize that mortality and morbidity are key economic drivers in nursery feeding programs and may outweigh differences in growth performance alone. As in the present study, removals and mortality did not differ statistically among treatments their impact was not incorporated into the economic analysis. However, the authors recognize that mortality and morbidity can substantially influence the economic optimization of nursery feeding programs, and larger studies may be required to more fully characterize these effects.

It was expected that the use of complex diets for a longer period may reduce mortality and removals, as the highly digestible ingredients with low concentrations of antinutritional factors improve nutrient utilization and support gastrointestinal health. In addition, greater feed intake associated with complex diets provides the nutrients necessary to sustain growth and immune function (Carnino et al. 2025). The combination of improved digestibility and higher intake reduces the likelihood of fall-behind pigs, which in turn lowers the incidence of morbidity and the need for removals (Koo et al. 2017). However, despite sufficient physiological and nutritional support for reductions in mortality and removals with the use of complex diets, similar to the present results, other studies have also reported no differences in removals or mortality associated with feeding duration of complex diets (Leliveld et al. 2013; González-Solé et al. 2024). In Exp. 1, the absence of health challenges likely explains why no pigs were removed or died during the trial. In Exp. 2, although pigs originated from a PRRS-positive flow, the intensive water medication program may have contributed to the low mortality and removal rates observed across all treatments. Furthermore, the lack of a significant treatment effect on CV suggests that the use of simple nursery feed budgets did not increase the number of fall-behind pigs that were not removed from the pen.

An alternative explanation for the lack of significant differences in removals and mortality in Exp. 2 relates to experimental power and sample size. With 15 replications per treatment, the estimated statistical power to detect differences in removals and mortality was approximately 11%. Power calculations indicated that approximately 184 replications per treatment would have been required to achieve 80% power to detect differences of the magnitude observed in the current experiment. Low statistical power increases the probability of Type II error (Kaps and Lamberson 2017), particularly when evaluating variable outcomes such as mortality and removals under commercial conditions. Similar limitations when evaluating mortality in swine experiments under commercial conditions have been previously discussed in the literature (Gebhardt et al. 2020).

Collectively, these results indicate that reducing nursery feed budgets did not increase removals, mortality, or BW variability under the conditions of this study. However, given the low statistical power for removals and mortality, these findings should be interpreted with caution. Future studies with substantially greater replication are warranted to validate that reductions in nursery feed budgets do not adversely affect removals or mortality rates under commercial production systems.

In conclusion, under both university and commercial conditions, reducing phase 1 and 2 feed budgets did not affect overall pig growth performance; however, reducing the quantity of phase 1 and 2 diets reduced feed cost and feed cost per unit of gain, leading to a greater IOFC for pigs fed the low-budget program. Therefore, the strategic adjustment of budgets for the first dietary phases could be a tool to improve economic return without compromising growth performance.

## Abbreviations

ADFI: average daily feed intake
ADG: average daily gain
BW: body weight
DM: fecal dry matter
IOFC: income over feed cost
PRRS: porcine reproductive and respiratory syndrome
SID: standardized ileal digestibility
